# Insulin response in peripheral tissues in animals chronically treated with aqueous extract of Hibiscus sabdariffa L.

**DOI:** 10.1186/1758-5996-7-S1-A117

**Published:** 2015-11-11

**Authors:** Carolina Abreu Miranda, Tatiele Estefâni Schönholzer, Betina Beatriz Mielke, Dener Lucas Araújo dos Santos, Dionys de Souza Almeida, Gustavo Tadeu Volpato, Kleber Eduardo de Campos

**Affiliations:** 1 Universidade Federal de Mato Grosso, Barra do Garças, Brazil

## Background

In 21st century, observed changes in dietary and nutritional standards, such as high consumption of carbohydrates and lipids, lead to an increased search for effective alternatives to treatment of metabolic disorders associated with this type of diet.

## Objective

To evaluate the effects of aqueous extract of Hibiscus sabdariffa L. (HS) by chronic treatment on the ability of peripheral tissues to promote glucose uptake in experimental animals with mild hyperglycemia.

## Materials and methods

Male Wistar rats were primary divided into two groups: Control (C) received standard chow and water ad libitum; Fructose (F) received standard chow and water containing 7% of fructose ad libitum. At 90 days of age they were one more time re-divided into 4 groups: Control treated with water (C, n=7); Control treated with HS (CHib, n=6); Fructose treated with water (F, n=8); Fructose treated with HS (FHib, n=9). Treatment with HS was daily for 4 weeks at a dose of 800 mg/kg. After treatment period, all rats were submitted to insulin tolerance tests (ITT) with two types of insulin (intermediary and short acting) in order to assess the peripheral tissues in response the action of insulin. Data obtained was estimated glucose uptake index (GUI). All data statistically analyzed with 5% significance.

## Results

TTI performed with insulin NPH (intermediary) show that only C group decreased glycaemia at timepoint 15′ compared to fasting glycemia (0′). The analyses between groups shows that CHib animals increased at timepoint 15′ and F group decreased at fasting and 5′ timepoints. The GUI data showed the F group had lowered the glucose uptake capacity compared to C group. The TTI performed with insulin Lispro (short acting) showed that CHib group decreased glucose concentration at timepoint 15′. On the other hand, F and FHib groups decreased in 10′ and 15′ compared to its fasting glycemia. Evaluations between groups shows the CHib rats lowered the serum glucose at 15′, F rats decreased at timepoints 5′, ‘10’ and 15′ and finally the FHib rats decreased this biomarker at 10′ and 15′. GUI data for Lispo ITT shows the fructose treatment improved the glucose uptake when compared to control rats

## Conclusions

The type of time action of insulin may influence the peripheral tissue responses, inducing glycemic changes in the groups that received fructose, promoting more sensitivity to insulin Lispro action in these groups, which differs from what observed when used insulin NPH.

